# Supplementary material for: Flow Cytometric Analysis of Bone Marrow Particle Cells for Measuring Minimal Residual Disease in Multiple Myeloma
Source: Cancers (Basel). 2022 Oct 8;14(19):4937. doi: 10.3390/cancers14194937 (PMC9563644; doi:10.3390/cancers14194937)
Supplement: Supplementary file 1 [file cancers-14-04937-s001.zip › Figure S1.pdf]

BM

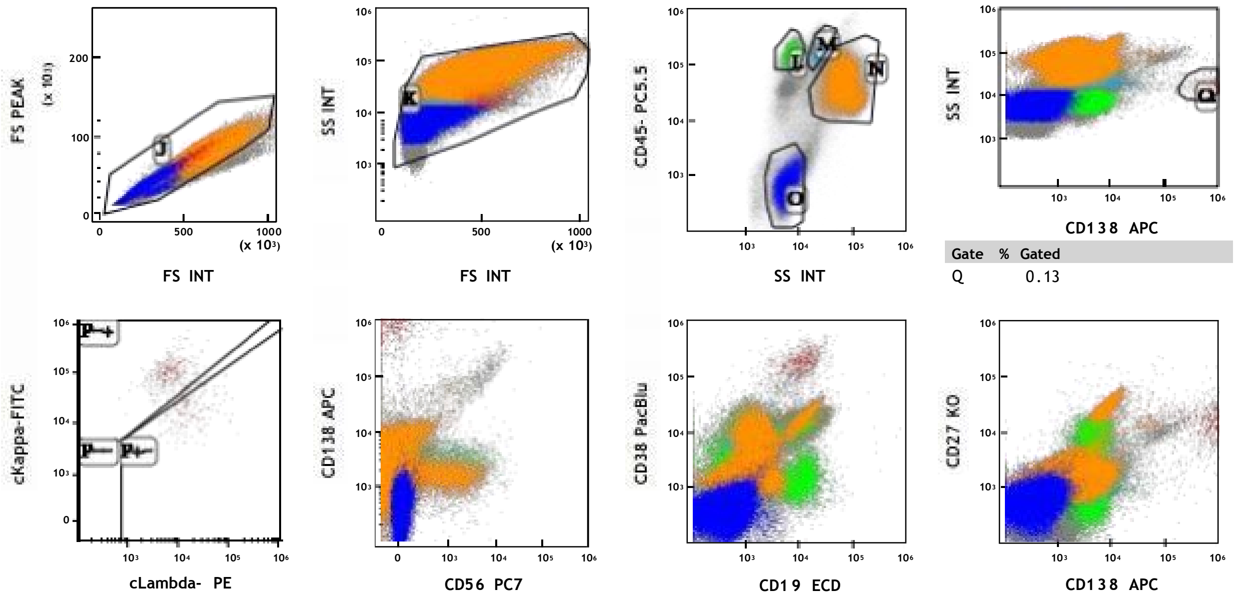

BMPL

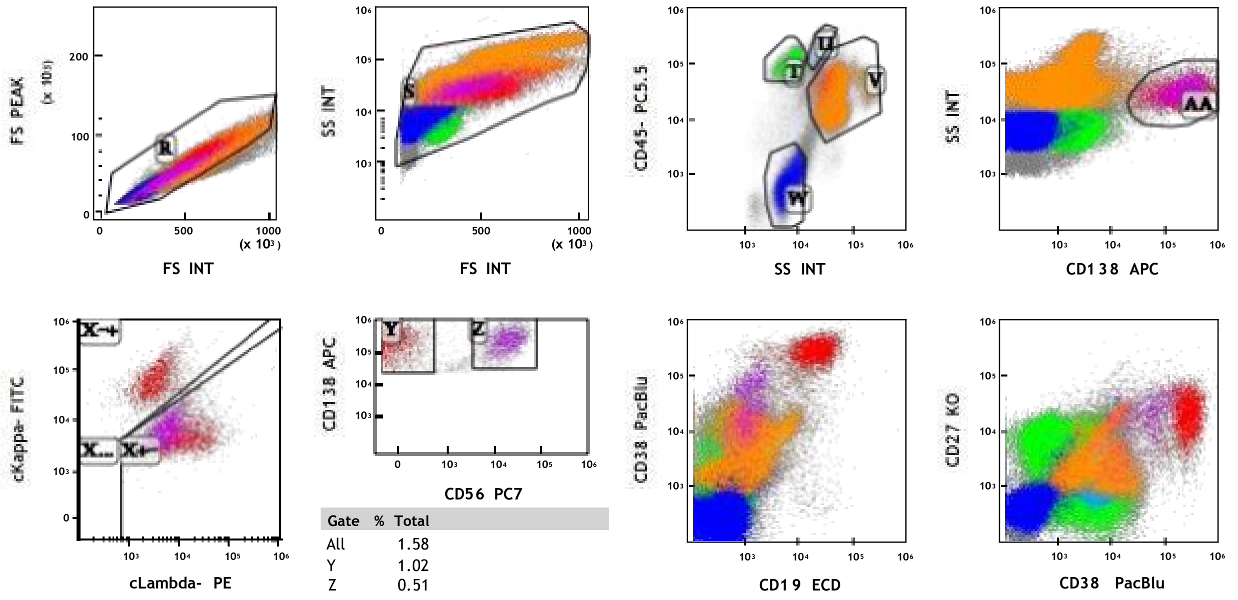

Supplementary Figure S1. Representative images of flow cytometry analysis for abnormal plasma cells (aPC) in patients at diagnosis. To show the significant differences between the two sample preparation methods (traditional BM samples vs BMPL samples)
