# Supplementary material for: Flow Cytometric Analysis of Bone Marrow Particle Cells for Measuring Minimal Residual Disease in Multiple Myeloma
Source: Cancers (Basel). 2022 Oct 8;14(19):4937. doi: 10.3390/cancers14194937 (PMC9563644; doi:10.3390/cancers14194937)
Supplement: Supplementary file 1 [file cancers-14-04937-s001.zip › Supplementary method.pdf]

Enrichment of bone marrow particle cells (BMPLs)

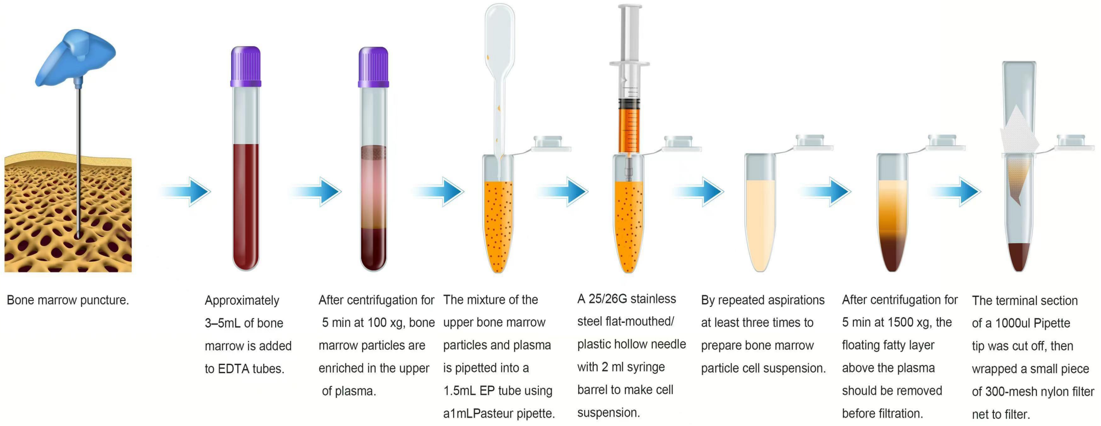

*S*tep 1: Approximately 3–5mL of bone marrow is re-aspirated after the bone marrow aspiration smear(the puncture needle need not be pulled out) and added to ethylenediamine tetraacetic acid(EDTA)-containing anticoagulant tubes. To obtain more particles, it is recommended to use a 20mL syringe during re-aspiration, and more bone marrow should be aspirated appropriately. If the tests cannot be completed in time, the tubes should be stored at 2-8 ℃. The tube is placed upright for 0.5– 1 h(not necessary), and then centrifuged for 5 min at 100 xg.

Step 2: The mixture (about 0.5 – 1.0 ml) of the upper BM particles and plasma is pipetted into a 1.5 ml EP tube. BM particles may also be added to pure normal saline in the EP tube.

Step 3: By repeated aspirations at least three times with a connecting device of 25/26G stainless steel flat-mouthed/plastic hollow needle and 2 ml syringe barrel, the upper bone marrow particles and plasma in Step 2 was mixed to prepare bone marrow particle cell suspension. If bone marrow particles cannot pass through a 25/26G needle, a 23G needle is to be used first.

Step 4: After centrifugation for 5 min at 1500 xg, the floating layer above the plasma is removed and the remaining plasma is mixed with cells at the bottom and then filtered with a 300-mesh nylon filter net to remove any residual fibrous components. If sufficient bone marrow particles have been obtained in step 2, the suspension may be filtered without centrifugation to simplify the process. The sample is then ready for creating bone marrow smear films, for flow cytometry(FCM) analysis, and for

polymerase chain reaction/fluorescence in situ hybridization/next-generation sequencing(PCR/FISH/NGS)analysis. Bone marrow particle cell suspension should be completed tests as soon as possible(within 6 hours). In general, it is not recommended to keep in cold storage.
