# Supplementary material for: Flow Cytometric Analysis of Bone Marrow Particle Cells for Measuring Minimal Residual Disease in Multiple Myeloma
Source: Cancers (Basel). 2022 Oct 8;14(19):4937. doi: 10.3390/cancers14194937 (PMC9563644; doi:10.3390/cancers14194937)
Supplement: Supplementary file 1 [file cancers-14-04937-s001.zip › Table S1.pdf]

Supplemental Table S1. Antibodies used in this study and their source.

Marker	Fluorochrome conjugate	Manufacturer	Catalogue number
**CD45**	PC5.5	Beckman Coulter	A62835
**CD138**	APC	Beckman Coulter	A87787
**CD38**	PacB	Beckman Coulter	B92396
**CD19**	ECD	Beckman Coulter	A07770
**CD27**	KrO	BioLegend	302836
**CD56**	PC7	Beckman Coulter	A21692
**cKappa**	FITC	Dako	F0434
**cLambda**	PE	Dako	R0437

Abbreviations:CD:cluster of differentiation; PC5.5:phycoerythrin-cyanine 5.5; APC:allophycocyanin; PacB:pacific blue; ECD:phycoerythrin-texas red; KrO:krome orange; PC7:phycoerythrin-cyanine 7; FITC:fluorescein isothiocyanate; PE:phycoerythrin; cKappa:cytoplasmic kappa (κ); cLambda:cytoplasmic lambda (λ).
