# Supplementary material for: Flow Cytometric Analysis of Bone Marrow Particle Cells for Measuring Minimal Residual Disease in Multiple Myeloma
Source: Cancers (Basel). 2022 Oct 8;14(19):4937. doi: 10.3390/cancers14194937 (PMC9563644; doi:10.3390/cancers14194937)
Supplement: Supplementary file 1 [file cancers-14-04937-s001.zip › Table S2.pdf]

Supplemental Table S2. The characteristics of patients with either minimal residual disease positive or negative bone marrow samples under treatment, measured with bone marrows particle cell (BMPL) enriched samples and bone marrows (BM) samples not enriched.

Patient's parameters	BMPL enriched samples (n=60)		BM samples (n=74)	
	MRD^+ (n=44)	MRD^- (n=16)	MRD^+ (n=44)	MRD^- (n=30)
Disease setting (%)				
post CTx	25 (57)	1 (6)	23 (52)	7 (23)
post auto-SCT	19 (43)	15 (94)	21 (48)	23 (77)
Median age, years (range)	59 (34-78)	57 (39-68)	59 (34-78)	55 (41-66)
Sex (%)				
Female	17 (39)	5 (31)	21 (48)	12 (40)
Male	27 (61)	11 (69)	23 (52)	18 (60)
Durie & Salmon (%)				
I	0 (0)	3 (19)	0 (0)	3 (10)
II	14 (32)	1 (6)	14 (32)	3 (10)
III	30 (68)	12 (75)	30 (68)	24 (80)
A/B	36 (82)/8 (18)	8 (50)/8 (50)	33 (75)/11 (25)	19 (63)/11 (37)
R-ISS (%)				
I	1 (2)	2 (12)	2 (5)	2 (7)
II	23 (52)	6 (38)	22 (50)	11 (36)
III	20 (46)	8 (50)	20 (45)	17 (57)
%PC of aspirate smears (range)	2 (0-14)	0.5 (0-8)	2 (0-21)	1 (0-12)
Cytogenetics [a] (%)				
High-risk	13 (30)	5 (31)	14 (32)	8 (27)
Intermediate-risk	7 (16)	1 (6)	6 (14)	3 (10)
Standard-risk	18 (41)	10 (63)	19 (43)	14 (47)
Missing	6 (13)	0 (0)	5 (11)	5 (16)
MM type (%)				
IgG	24 (55)	8 (50)	24 (55)	16 (53)
IgA	16 (36)	3 (19)	13 (30)	7 (24)
IgD	1 (2)	1 (6)	1 (2)	1 (3)
Light chain only	2 (5)	4 (25)	5 (11)	6 (20)
Biclonal	1 (2)	0 (0)	1 (2)	0 (0)
Light chain (%)				
Kappa	17 (39)	7 (44)	23 (52)	11 (37)
Lambda	26 (59)	9 (56)	20 (46)	19 (63)
Biclonal	1 (2)	0 (0)	1 (2)	0 (0)
Remission [b] (%)				
CR	7 (16)	7 (44)	8 (18)	9 (30)
VGPR	30 (68)	9 (56)	29 (66)	18 (60)

PR	6 (14)	0 (0)	5 (11)	3 (10)
SD	1 (2)	0 (0)	2 (5)	0 (0)
MM Progression (%)				
Yes	9 (20)	1 (6)	9 (20)	4 (14)
No	35 (80)	15 (94)	35 (80)	26 (86)
Vital status (%)				
Dead	3 (7)	0 (0)	4 (9)	0 (0)
Alive	41 (93)	16 (100)	40 (91)	30 (100)

a: According to Mayo Stratification of Myeloma and Risk-Adapted Therapy (mSMART) Consensus Guidelines.

b: According to International Myeloma Working Group criteria.

Abbreviation: BM: bone marrow; BMPL: bone marrow particle cell; MRD: minimal residual disease; post-CTx: after standard chemotherapy; post-ASCT: after autologous stem cell transplantation; PC: plasma cells; CR: complete response; VGPR: very good partial response; PR: partial response; SD: stable disease.
